# Natural variation in neuraminidase activity influences the evolutionary potential of the seasonal H1N1 lineage hemagglutinin

**DOI:** 10.1093/ve/veae046

**Published:** 2024-06-19

**Authors:** Tongyu Liu, William K Reiser, Timothy J C Tan, Huibin Lv, Joel Rivera-Cardona, Kyle Heimburger, Nicholas C Wu, Christopher B Brooke

**Affiliations:** Department of Microbiology, University of Illinois at Urbana-Champaign, Urbana, IL 61801, USA; Department of Microbiology, University of Illinois at Urbana-Champaign, Urbana, IL 61801, USA; Center for Biophysics and Quantitative Biology, University of Illinois at Urbana-Champaign, Urbana, IL 61801, USA; Carl R. Woese Institute for Genomic Biology, University of Illinois at Urbana-Champaign, Urbana, IL 61801, USA; Department of Biochemistry, University of Illinois at Urbana-Champaign, Urbana, IL 61801, USA; Department of Microbiology, University of Illinois at Urbana-Champaign, Urbana, IL 61801, USA; Department of Microbiology, University of Illinois at Urbana-Champaign, Urbana, IL 61801, USA; Center for Biophysics and Quantitative Biology, University of Illinois at Urbana-Champaign, Urbana, IL 61801, USA; Carl R. Woese Institute for Genomic Biology, University of Illinois at Urbana-Champaign, Urbana, IL 61801, USA; Department of Biochemistry, University of Illinois at Urbana-Champaign, Urbana, IL 61801, USA; Carle Illinois College of Medicine, University of Illinois at Urbana-Champaign, Urbana, IL 61801, USA; Department of Microbiology, University of Illinois at Urbana-Champaign, Urbana, IL 61801, USA; Carl R. Woese Institute for Genomic Biology, University of Illinois at Urbana-Champaign, Urbana, IL 61801, USA

**Keywords:** influenza virus, antigenic evolution, epistasis, mutational fitness landscape, HA–NA balance

## Abstract

The antigenic evolution of the influenza A virus hemagglutinin (HA) gene poses a major challenge for the development of vaccines capable of eliciting long-term protection. Prior efforts to understand the mechanisms that govern viral antigenic evolution mainly focus on HA in isolation, ignoring the fact that HA must act in concert with the viral neuraminidase (NA) during replication and spread. Numerous studies have demonstrated that the degree to which the receptor-binding avidity of HA and receptor-cleaving activity of NA are balanced with each other influences overall viral fitness. We recently showed that changes in NA activity can significantly alter the mutational fitness landscape of HA in the context of a lab-adapted virus strain. Here, we test whether natural variation in relative NA activity can influence the evolutionary potential of HA in the context of the seasonal H1N1 lineage (pdmH1N1) that has circulated in humans since the 2009 pandemic. We observed substantial variation in the relative activities of NA proteins encoded by a panel of H1N1 vaccine strains isolated between 2009 and 2019. We comprehensively assessed the effect of NA background on the HA mutational fitness landscape in the circulating pdmH1N1 lineage using deep mutational scanning and observed pronounced epistasis between NA and residues in or near the receptor-binding site of HA. To determine whether NA variation could influence the antigenic evolution of HA, we performed neutralizing antibody selection experiments using a panel of monoclonal antibodies targeting different HA epitopes. We found that the specific antibody escape profiles of HA were highly contingent upon NA background. Overall, our results indicate that natural variation in NA activity plays a significant role in governing the evolutionary potential of HA in the currently circulating pdmH1N1 lineage.

## Introduction

Seasonal influenza A viruses (IAVs) undergo rapid evolution in the human population, especially within the surface glycoproteins hemagglutinin (HA) and neuraminidase (NA) (Han et al. 2023, Kistler and Bedford 2023). Substitutions within neutralizing antibody epitopes on HA, particularly in residues surrounding the receptor-binding site (RBS), are thought to be the primary driver of viral antigenic evolution and the associated declines in vaccine efficacy (Smith et al. 2004, Koel et al. 2013, Bedford et al. 2014, Chambers et al. 2015). Every 2–8 years, antigenically novel variants sweep across the globe, replacing previously circulating clades in a process known as an antigenic cluster transition (Smith et al. 2004, Koelle et al. 2006, Koel et al. 2013). These cluster transitions often coincide with elevated influenza-associated mortality rates (Greene et al. 2006, Chambers et al. 2015, Cobey and Hensley 2017).

Given the high mutation rates of IAVs observed *in vitro* (Parvin et al. 1986, Suárez-López and Ortín 1994, Brooke 2017, Pauly et al. 2017), it remains unclear why antigenic cluster transitions do not occur more frequently. Numerous factors including narrow transmission bottlenecks, deleterious mutation load, and inter- and intra-segment epistatic effects likely all contribute to limiting the ability of the virus to explore novel antigenic space (Koelle and Rasmussen 2015, Wu and Wilson 2017, McCrone et al. 2018, Wu et al. 2018, 2020a). Identifying the specific factors that influence the emergence of antigenically novel variants is critical for improving the vaccine strain selection process and informing next-generation vaccine design.

During viral entry, HA forms multivalent bonds with glycoconjugates that contain terminal sialic acid residues (Benton et al. 2015, Guo et al. 2018, Sieben et al. 2020). These sialic acid receptors are common in the mucosal lumen, and NA plays an essential role in dissociating the virion from sialylated factors in the extracellular space or non-functional cell surface sialic acids to facilitate entry (McAuley et al. 2019). During virion release, NA liberates newly assembled virions from the sialic acids on the cell, preventing entrapment and aggregation and promoting virus spread (Palese et al. 1974, Palese and Compans 1976, Guo et al. 2023). Thus, suboptimal HA–NA balance can affect the efficiency of both entry and release (McAuley et al. 2019). Numerous studies have suggested the importance of maintaining HA–NA functional balance during antigenic evolution, the evolution of drug resistance, and the emergence and adaptation of the 2009 H1N1 pandemic strain (Hensley et al. 2011, Ginting et al. 2012, Matsuzaki et al. 2014, Neverov et al. 2015, Wang et al. 2021).

We recently used the mouse-adapted H1N1 strain A/Puerto Rico/8/1934 (PR8) to demonstrate that the evolutionary potential of HA can be significantly constrained by epistatic interactions with NA (Liu et al. 2022), suggesting that the need to maintain HA–NA functional balance may restrict antigenic variant emergence potential (Kosik and Yewdell 2019, McAuley et al. 2019, de Vries et al. 2020). To assess the relevance of this phenomenon for recent seasonal H1N1 viruses, we generated recombinant viruses encoding the NA segments from H1N1 vaccine strains isolated between the pandemic lineage emergence in 2009 and 2019. We compared the NA activity phenotypes of these different N1 genotypes and asked how natural variation in NA activity altered both the mutational fitness landscape of HA1 by deep mutational scanning (DMS) and the genetic pathways of escape from neutralizing antibody selection. Our results demonstrate that epistatic interactions with NA can play a significant role in influencing HA antigenic evolution in the seasonal H1N1 lineage.

## Results

### Variation in NA activity within the pdmH1N1 lineage

**Figure 1.**
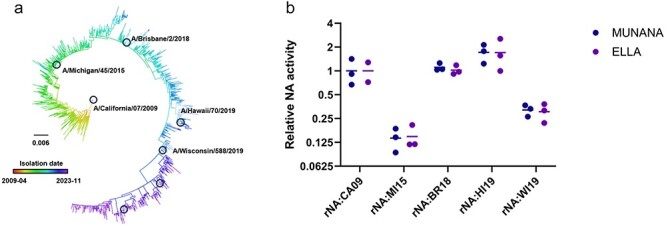
Relative NA activity varies across the pdmH1N1 lineage. (a) Phylogenic tree of the pdmH1N1 lineage NA segment with branch lengths in units of divergence (generated by Nextstrain (Hadfield et al. 2018) and visualized in FigTree (FigTreehttp://tree.bio.ed.ac.uk/software/figtree/)). Vaccine strains marked with circles, strains used in this study are labeled with strain name. (b) Relative, virion-associated NA activities of recombinant viruses encoding the indicated NA genes in Cal09 backbone measured by either MUNANA or ELLA. Results were normalized based on genome equivalents of the NP segment as measured by qPCR.

The pdmH1N1 NA gene accumulates ∼4 × 10^−3^ nucleotide substitutions per codon year, partially due to immune pressure against NA in the population (Hadfield et al. 2018, Yasuhara et al. 2019, Powell et al. 2020, Kistler and Bedford 2023). To capture the evolution of the NA gene from its emergence into humans in 2009 to 2019, we selected five vaccine strains chosen by the WHO over this time period: A/California/07/2009 (CA09), A/Michigan/45/2015 (MI15), A/Brisbane/2/2018 (BR18), A/Hawaii/70/2019 (HI19), and A/Wisconsin/588/2019 (WI19) (Fig. 1a). Twenty-six amino acid substitutions differentiate the NA proteins of WI19 and CA09. We used reverse genetics to generate recombinant viruses that encode the NA genes from each of the five vaccine strains, with the remaining seven segments coming from CA09. Note that a single substitution, HA:G158E, repeatedly emerged to high frequency in working stocks of CA09, likely because it enhanced fitness in the MDCK-SIAT1 cells we used. To minimize the potential for strong selection on this substitution confounding our downstream experiments, we fixed G158E in the CA09 HA segment of all viruses used in this study.

We compared the NA activities of these viruses against the soluble NA substrate 2′-(4-methylumbelliferyl)-α-*D-N*-acetylneuraminic acid (MUNANA) and against surface-immobilized fetuin in the enzyme-linked lectin assay (ELLA) (Fig. 1b). We observed a nearly ten-fold range in virion-associated NA activity across the NA genes tested. Notably, the two vaccine strains isolated in 2019, HI19 and WI19, exhibited a roughly four-fold difference in NA activity relative to each other, demonstrating how multiple NA genotypes with distinct NA activity phenotypes may co-circulate in a given year.

### Variation in NA background has little effect on the overall mutational fitness landscape of the pdm2009 lineage HA1

In our previous study, we observed that decreasing NA activity was associated with a global shift in the mutational robustness of HA, as measured by DMS (Liu et al. 2022). To determine if the natural variation in NA activity we observed across the pdm2009 lineage could have the same effect, we used DMS to introduce all possible single amino acid substitutions in the HA1 subunit of the CA09 HA gene and compare the fitness effects of each substitution across different NA backgrounds.

We generated a hypermutagenized plasmid library containing all possible single amino acid substitutions in the HA1 subunit by degenerate polymerase chain reaction (PCR) and rescued triplicate virus libraries for three different NA genes, CA09, HI19, and WI19, all in an identical CA09 backbone by reverse genetics (rNA:CA09, rNA:HI19, and rNA:WI19). We chose HI19 and WI19 as comparators because they (i) represent two NA clades (primarily associated with HA Clades 5a.1 and 5a.2) that co-circulated from 2019 to 2022, and (ii) exhibit a roughly four-fold range in relative NA activity in our assays.

For comparison, we also generated triplicate rNA:CA09 virus libraries in the presence of the NA inhibitor zanamivir at a concentration we measured to inhibit 90 per cent of NA activity (rNA:CA09 + NAI) (Supplementary Fig. S1). All virus libraries were passaged once for 36 h in MDCK-SIAT1 cells, starting at a low multiplicity of infection (MOI; 0.05 TCID50/cell). We collected viral supernatants and sequenced the post-passage virus populations along with the input plasmid library and wild-type plasmid by barcoded subamplicon sequencing (Wu et al. 2014, Doud and Bloom 2016).

Out of 6846 possible mutations within the HA1 subunit, 6557 mutations had sufficient sequencing depth with good quality and were included in downstream analysis. We calculated relative fitness scores (representing relative growth advantages in our cell culture system) for each substitution based on the changes in frequencies between the plasmid library and the post-passage population. Given that silent mutations are likely neutral and nonsense mutations are likely lethal, we normalized relative fitness scores of all substitutions based on the mean relative fitness scores of all silent and nonsense mutations in each sample so that the mean silent mutation value was set at a score of 1 and the mean nonsense mutation value would have a score of 0. The normalized fitness scores of each mutation showed a high degree of correlation between replicate passages (Pearson correlations ranged from 0.72 to 0.81, Supplementary Fig. S2), suggesting that our relative fitness effect estimates were reproducible across replicate populations.

**Figure 2.**
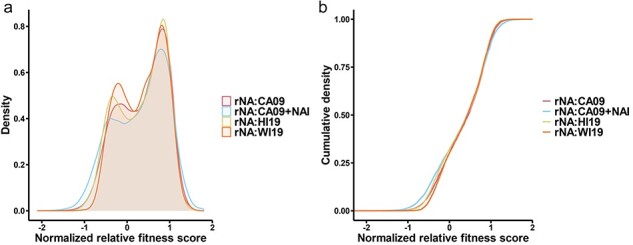
Changes in NA background have minimal impact on the overall mutational fitness landscape of HA1. (a) The normalized relative fitness distribution and (b) cumulative density distribution of all missense mutations within HA1 subunit for each virus genotype tested as well as the CA09 + NAI control.

The normalized relative fitness score distributions in the different NA backgrounds were bimodal, with a primary peak around a score of 1 and a smaller peak around the score of 0 (Fig. 2). In contrast with our previous results obtained using PR8 (Liu et al. 2022), the fitness score distributions were similar across the different NA backgrounds and the CA09 + NAI control, suggesting that the range of NA activities tested here had little effect on the overall mutational robustness of HA1 in the pdm2009 lineage. The means of the average fitness scores from the different NA backgrounds were statistically significant (*P* < 0.001 by the Wilcoxon matched-pairs signed rank test); however, so were the differences between technical replicates for the individual groups, suggesting that these differences primarily reflect technical noise. Altogether, these data indicate that the NA genotype has a minimal effect on the global mutational robustness of HA1.

### Residues involved in receptor binding exhibit epistasis with NA

To determine whether changes in NA activity were associated with epistatic effects on specific HA residues, we performed linear regressions using the fitness score of each substitution as the dependent variable and the relative NA activity as the independent variable for the different NA backgrounds. We calculated the coefficient value of each substitution as the slope of the best-fit linear regression. Substitutions that exhibited higher relative fitness in the context of higher NA activity had positive coefficient values, and those with higher relative fitness in the context of lower NA activity had negative coefficient values (Supplementary Fig. S3). The absolute value of the coefficient quantifies the sensitivity of a given substitution to the epistatic influence of NA.

**Figure 3.**
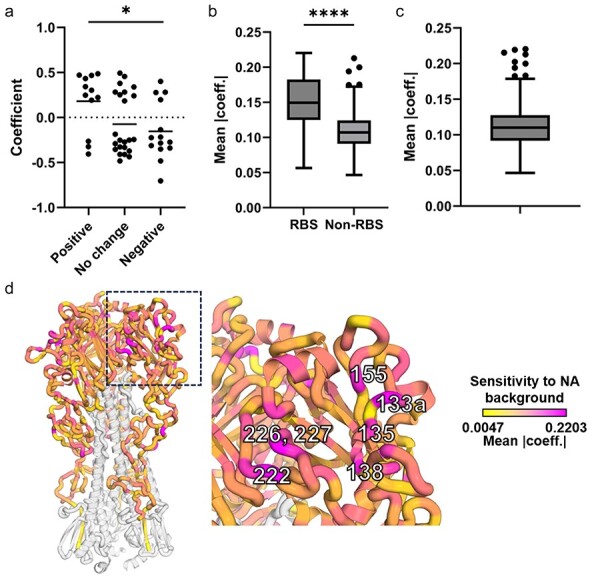
Epistatic interactions with NA are most pronounced in RBS-associated residues. (a) The coefficient values (*P* < .001) of different charges change on the HA surface, single asterisk indicates *P* < .05 generated by Mann–Whitney test. Substitutions with *P* > .001 are not shown. *P*-value is also <.05 by Mann–Whitney test when all substitutions are included. (b) Comparison of MAC values of RBS-associated residues (defined as residues in the 130-loop, 190-helix, 220-loop, or receptor-binding pocket) versus the other residues in HA1, four asterisks indicates *P* < .0001 generated by Mann–Whitney test. (c) The Tukey box and whisker plot of MAC for all residues, outliers are shown as dots that are higher than 75th percentile plus 1.5 times the interquartile range (IQR). (d) Structure of HA (PDB: 3UBQ (Xu et al. 2012)) colored by MAC value. The residue numbers on the RBS that were outlier high MAC values identified in (c) are labeled.

Given that both sialic acids and the exterior of the plasma membrane are negatively charged, we predicted that substitutions that increased positive charge on the HA surface would enhance attachment to the cell surface and sialic acids and thus would exhibit higher relative fitness in the context of higher NA activity. Similarly, we predicted that negative charge changes would be better tolerated in reduced NA activity backgrounds. Consistent with this, we observed significantly more positive charge changes with positive coefficient values than negative charge changes (Fig. 3a).

To quantify the overall epistatic influence of NA over each HA1 residue, we calculated the mean of the absolute coefficient (MAC) values for each position by determining the absolute coefficient values of all nineteen possible missense substitutions based on a linear regression of the fitness scores against the measured NA activity for each NA background tested. A higher MAC value for a given residue indicated a greater influence of NA activity on the combined fitness effects of all possible substitutions at that position.

We hypothesized that substitutions at RBS-associated residues are more likely to have pronounced effects on receptor-binding avidity than substitutions distal from the RBS and thus should be subject to stronger epistatic interactions with NA. We observed that the RBS residues exhibited higher MAC values than non-RBS residues (Fig. 3b). When we examined the overall distribution of MAC values across HA1, we identified residues 49, 133a, 135, 138, 155, 222, 226, 227, and 291 (based on the H3 structure-based numbering system, 133a indicates a position inserted between residues 133 and 134 based on the alignment to H3 Burke et al. 2014) as outliers, indicating that they are particularly sensitive to NA phenotype (Fig. 3c). With the exceptions of residues 49 and 291, all of these NA-sensitive residues are indirectly or directly adjacent to the RBS (Fig. 3d), demonstrating that NA epistatic effects are concentrated in and around the RBS.

### Specific mutational pathways of HA escape from neutralizing antibody selection are contingent upon NA genotype

We next asked whether different NA backgrounds supported the emergence of distinct HA escape variants in the presence of identical neutralizing anti-HA monoclonal antibodies (mAbs), as we recently observed for the lab-adapted PR8 strain (Liu et al. 2022). We used a panel of mAbs targeting distinct epitopes on HA (Table 1).

**Table 1.** mAbs used in the selection experiments.

Antibody	Epitope	Position	Reference
EM4-C04	Sa	HA head, upper RBS	O’Donnell et al. 2012, Matsuzaki et al. 2014
2A05	Ca1	HA head, lower RBS	Guthmiller et al. 2021
2C05	Ca2	HA head, lower RBS	Guthmiller et al. 2021
CR9114	Stem	HA stem	Dreyfus et al. 2012

For each mAb, we determined the saturating neutralization concentration, defined as the lowest mAb concentration that maximized the inhibition of virus yield under the same experimental conditions (virus population size, volume, etc.) as the subsequent selection experiments (Supplementary Fig. S4). For each mAb, we incubated 10^7^ TCID50 of each recombinant virus (pooled from three independent virus rescue transfections) with each mAb at its saturating neutralization concentration in six replicates. We used starting populations of 10^7^ TCID50 to maximize representation of all possible single-nucleotide substitutions in starting populations. This was based on the assumption that each replicated genome contains an average of two random nucleotide substitutions (Pauly et al. 2017), meaning that every possible single nucleotide mutation would occur ∼370 times on average within a starting viral population of 10^7^ TCID50 (ignoring the effects of variation in relative fitness and substitution type preferences of the viral replicase; Brooke 2017). We infected cells with virus/antibody mixtures and collected virus supernatants at 24 h post-infection for next-generation sequencing (NGS) to identify putative escape variants. If viral titers were insufficient (<10^4^ TCID50/ml) for generating high-quality sequencing libraries, we performed a second passage in the presence of the mAbs.

**Figure 4.**
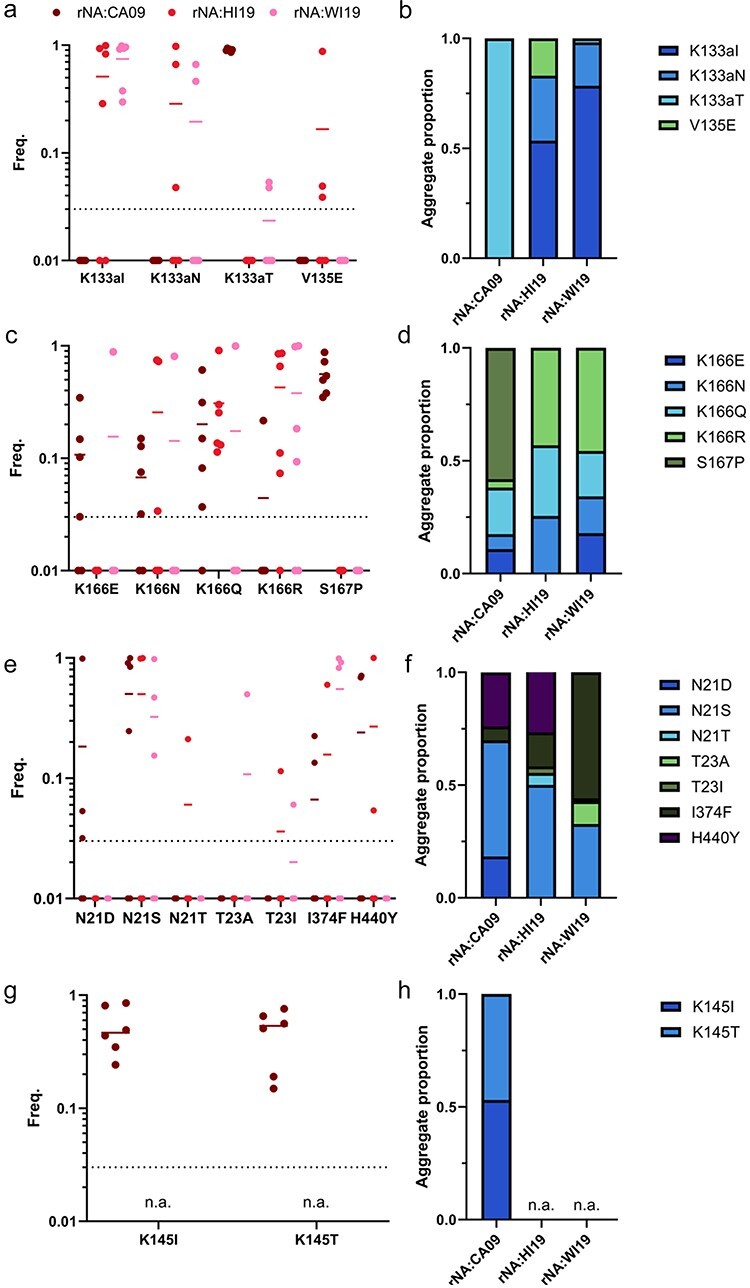
Specific mutational pathways of anti-HA antibody escape are contingent on NA activity. HA substitutions that emerged under mAb selection in different NA backgrounds. (a, c, e, and g) Frequencies of major substitutions (>3 per cent in a given replicate and >10 per cent in at least one replicate) detected under selection with (a) EM4-C04, (c) 2A05 (e) CR9114 and (g) 2C05 (n. a., not assayed due to no surviving virus.), in the indicated NA backgrounds [each dot represents the frequency in a single selection replicate (*n* = 6), except for CR9114 with rNA:WI19 (*n* = 4), 2A05, and CR9114 with rNA:WI19 (*n* = 5)]. (b, d, f, and h) The aggregate proportions of each indicated substitution summed across all replicates for each NA background.

Based on our DMS results, we hypothesized that RBS-proximal epitopes would be more sensitive to changes in NA background. For the Sa-specific mAb EM4-C04 (Wrammert et al. 2011, O’Donnell et al. 2012), K133aT was the dominant HA substitution that emerged in the rNA:CA09 background, while K133aI and K133aN were the major substitutions that emerged in rNA:HI19 and rNA:WI19 (Fig. 4a and b). Note that we identified K133a as one of the NA-sensitive residues by DMS (Fig. 3). K133aN first began to emerge during the 2018–19 flu season and swept to global dominance by 2023, raising the possibility that the emergence of this antigenic variant at the global scale may have depended in part on the emergence of suitable NA genotypes (Supplementary Fig. S5).

Under selection with the Ca1-specific mAb 2A05, S167P was the major substitution found in rNA:CA09 and K166R was the major substitution found in the other two NA backgrounds (Fig. 4c, d). Note that the escape profiles of rNA:HI19 and rNA:WI19 for both EM4-C04 and 2A05 are more similar to each other than to rNA:CA09, despite exhibiting a greater difference in virion-associated NA activity (Fig. 1b). This suggests that our NA activity assays may not capture all features of NA relevant for influencing HA fitness in our system.

To compare with escape from head-targeting mAbs, we performed selections using the stem-targeting mAb CR9114 (Dreyfus et al. 2012). We observed the emergence of substitutions at N21, T23, I374, and H440, all located in or under the HA stem epitope (Fig. 4e and f). N21S emerged to >80 per cent in one or more replicates for all three NA backgrounds examined. I374F (I45F, HA2 numbering) also repeatedly emerged to high frequency (>10 per cent) in one or more replicates across all NA backgrounds, suggesting that some anti-stem mAb escape pathways may be less constrained by epistatic interactions with NA than what we observe with RBS-proximal residues. We did observe some stem escape variants that differed between NA backgrounds, including N21D, which was observed in multiple rNA:CA09 replicates, but not with rNA:HI19 or rNA:WI19, and H440Y (H111Y, HA2 numbering), which emerged in multiple rNA:CA09 and rNA:HI19 replicates, but not rNA:WI19. Interestingly, H440Y is not surface exposed; it is located underneath the stem epitope, adjacent to I374. This suggests that substitutions at buried residues may trigger conformational shifts within the stem epitope sufficient to mediate escape.

**Figure 5.**
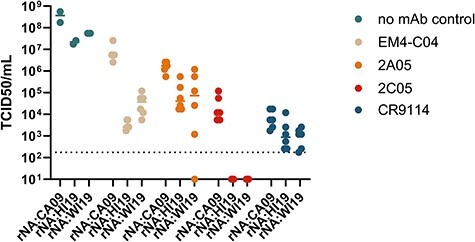
Virus titer post-antibody selection depends on NA background. TCID50 titers of viruses with different NA backgrounds (*n* = 6 for selection and *n* = 2 for no antibody control) following mAb selection.

Finally, under selection with the Ca2-specific mAb 2C05, K145I and K145T both emerged in the rNA:CA09 background; however, no virus could be recovered post-selection from the rNA:HI19 and rNA:WI19 backgrounds (Fig. 4g and h). This suggests that suboptimal HA–NA pairings may limit the potential for evolutionary rescue in the face of some anti-HA immune pressures. We also measured the titers of virus supernatants after the selection process and observed substantial differences depending on the mAb used. No mAb control titers for rNA:HI19 and rNA:WI19 were about ten-fold lower than that of rNA:CA09, indicating that rNA:HI19 and rNA:WI19 have a slight intrinsic growth disadvantage compared to rNA:CA09 in the absence of Ab pressure (Fig. 5). This cannot fully explain the much larger differences in post-selection titers for EM4-C04 and 2C05. This appears to be a mAb-specific phenomenon, as post-selection titer differences for 2A05 and CR9114 were comparable to no Ab controls. These results indicate that the NA background of the virus potentially influences the rate at which escape variants may emerge under antibody selection in an epitope-specific manner.

The specific dominant escape variants that emerged in different NA backgrounds under Ab selection could not be predicted based on the normalized relative fitness scores from the DMS experiment (Supplementary Fig. S6). For instance, S167P was the dominant variant that emerged in the rNA:CA09 background under 2A05 selection, despite having a lower relative fitness score than other possible escape variants. This discrepancy between the DMS results (which simply reflect relative growth advantages of individual substitutions) and the Ab escape results (which reflect a combination of mutation rates, initial population frequencies, growth advantage, and magnitude of Ab escape phenotype) illustrates how DMS-based approaches alone are insufficient for predicting the evolutionary trajectories of viral populations.

## Discussion

Our results here extend our earlier proof-of-principle experiments with the lab-adapted PR8 strain (Liu et al. 2022) to demonstrate that natural variation in NA within the pdm2009 H1N1 lineage can influence the specific genetic pathways taken by the virus to escape from anti-HA antibody pressure. These data clearly establish how the need to optimally tune the intimate functional partnership between HA and NA imposes distinct constraints on the evolutionary potential of recent seasonal H1N1 viruses.

When we examined the distribution of normalized mutational fitness effects within HA1 using DMS, we observed that the major peak was centered around a score of 1, consistent with a large fraction of potential substitutions being nearly neutral in our system. The findings contrast with the wild type (WT) PR8, where we observed the primary peak close to 0, indicating that most substitutions in that genetic background were highly deleterious or lethal (Liu et al. 2022). This suggests that the pdm2009 H1N1 lineage HA1 is more mutationally tolerant than that of PR8. Given that the degree of mutational robustness of a gene can influence its potential to access fitness peaks (Lauring et al. 2013), more work should be done to quantitatively compare mutational fitness effect distributions between different virus strains and across different host environments. Significant differences in these distributions could suggest that different viral strains vary in overall evolutionary potential.

Also contrasting with PR8, we did not observe any significant shifts in the fitness effect distribution between different NA backgrounds (Fig. 2). While we failed to detect a clear epistatic influence of NA over the global HA1 fitness landscape, we did detect strong epistatic effects for specific residues, primarily in or around the RBS. This is particularly important since RBS-proximal substitutions play a large role in driving antigenic cluster transitions (Koel et al. 2013). This observation, combined with our observation that the pdm2009 HA takes distinct mutational pathways to escape from neutralizing antibody pressure depending on the NA background, strongly suggests that the antigenic drift potential of seasonal H1N1 viruses is significantly influenced by epistatic interactions between HA and NA.

While our DMS analyses clearly identified residues with mutational fitness effects that correlated with NA activity, our Ab escape experiments did not reveal a simple relationship between measured NA activity and escape-variant emergence. For the head-specific mAbs EM4-C04, 2A05, and 2C05, we observed that rNA:HI19 and rNA:WI19 supported the emergence of more similar escape variants profiles to each other than either did to rNA:CA09. This does not line up with our NA activity data, where rNA:CA09 exhibited an intermediate phenotype in between those of rNA:HI19 and rNA:WI19. These data suggest that while NA can exert profound epistatic effects on the evolution of Ab resistance by HA, this effect cannot be simply explained by NA activity (at least as measured by the standard *in vitro* assays we use here). Future efforts should explore whether other NA features (e.g. receptor specificity, physical arrangements on virions, etc.) influence HA–NA epistasis.

We initially hypothesized that HA escape from stem Abs would not be significantly influenced by NA due to the distance between the stem epitope and the RBS. While the escape profiles for the three NA backgrounds did share some variants in common, namely N21S and I374F, rNA:WI19 differed from rNA:CA09 and rNA:HI19 both in the emergence of I374F at much higher frequencies and in the absence of H440Y. These results suggest that NA may influence antibody escape at the stem epitope, despite its distance from the RBS.

We note that H440Y emerged under selection with CR9114 in both rNA:CA09 and rNA:HI19 backgrounds, despite being surface-buried. H440Y is adjacent to the surface residue I374 where substitutions can convey resistance to CR9114 neutralization (as shown here and in a previous study (Wu et al. 2020b)). Mutations at this position also promote the escape of H5 from CR6261 (Throsby et al. 2008), a stem antibody encoded by the same germline gene GHV1-69 as CR9114. These data demonstrate how substitutions at residues buried underneath the stem epitope can mediate anti-stem Ab escape by inducing allosteric changes in the local structure that disrupt Ab binding.

Altogether our data highlight how epistatic interactions with NA constrain the evolutionary potential of HA in the seasonal pdm2009 H1N1 lineage. These findings indicate that a better understanding of HA–NA functional interactions in seasonal IAVs may help improve our ability to predict future evolutionary dynamics and enhance the accuracy of vaccine strain selection decisions. In practical terms, this will require more comprehensive genetic and phenotypic analysis of co-circulating HA and NA genotypes to determine whether HA–NA functional balance phenotypes can be inferred from sequence data and whether these phenotypes are predictive of the evolutionary success of specific sublineages.

## Materials and methods

### Cells and virus

HEK-293T and MDCK-SIAT1 cells were carried out in Minimum Essential Medium (MEM + GlutaMAX, Thermo Fisher Scientific) with 9.1 per cent fetal bovine serum (Avantor Seradigm Premium Grade Fetal Bovine Serum) in 37°C and 5 per cent CO_2_.

Expi293F cells (Gibco) were grown and maintained in Expi293 Expression Medium (Gibco) at 37°C, 5 per cent CO_2_, and 95 per cent humidity with shaking at 125 r.p.m.

Virus was first rescued by reverse genetics. Five hundred nanograms of each of eight plasmids that transcribe both positive- and negative-sense viral RNA were used to transfect HEK-293T cells in six-well plate by jetPRIME (Polyplus Transfection) according to the manufacturer’s protocol. Twenty-four hours post-transfection, the medium was replaced by infectious medium (MEM + 1 μg/ml TPCK-treated trypsin + 1 mM HEPES and 50 μg/ml gentamicin). MDCK-SIAT cells were co-seeded if necessary. The supernatant was then collected 48 h post-transfection and used to infect confluent MDCK-SIAT cells in six-well plates. For infection, MDCK-SIAT cells were first washed once with phosphate buffered saline (PBS) and the virus supernatant was added on top of the monolayer. After 1 h of inoculation in 37°C on shaker, the monolayer was washed again with PBS and infectious medium was added on the monolayer. The supernatant was collected 24–48 h post-infection as the seed stock of the virus. To generate the working stock of the virus, confluent T75 or T175 flask of MDCK-SIAT1 were infected with MOI of 0.0001 based on the TCID50 titer to avoid the generation of defective interference particle, and the supernatant was collected 48–72 h post-infection.

One adaptive mutation to our cell culture system on HA (HA:G158E) was constantly found in high frequency in the working stock and readily fixed after few passages. We decided to introduce this mutation into the virus as the new CA09 backbone that would otherwise unavoidably emerge in our experiment. The numbering of HA and NA is based on the alignment of H3N2 amino acid sequence (strain A/Hong Kong/1/1968 H3N2, UniProt: Q91MA7, Q91MA2 for HA and NA) without the localization signal peptide unless otherwise specified.

### TCID50 assay

Ninety-six-well plate was seeded a day prior to the experiment with 4–5 × 10^4^ MDCK-SIAT1 cells per well. On the day of the experiment, cells were washed with PBS once and 180 μl of infectious medium was added into each well. Twenty microliters of virus was added into the first four rows of first column and continued with 10-fold serial dilution. Between each dilution, tips were changed to avoid carrying-over. The plates were scored for the endpoint of cytopathic effect of each row 5–7 days post-infection. The TCID50 titer was calculated by Reed and Muench (1938) method.

### Quantification of virus particle concentration by RT-qPCR

To remove extracellular viral RNA, 140 μl virus supernatant was treated with 0.25 μg RNaseA for 30 min in 37°C and continued with viral RNA extraction (QIAamp Viral RNA Kits, Qiagen) following the manufacturer’s protocol. cDNA of the virus genome was generated by reverse transcription (Verso cDNA Synthesis Kit, Thermo Fisher Scientific) using MBTuni-12 (5′-ACGCGTGATCAGCAAAAGCAGG) as the primer (Zhou et al. 2009) and the virus RNA as the template. The cDNA was then used for qPCR (TaqMan Fast Advanced Master Mix, Thermo Fisher Scientific) detailed previously (Alnaji et al. 2021) by primers and probe targeting the NP segment, which generate less internal deletion based on our previous study (Alnaji et al. 2019, 2021). The relevant virus particle concentration (genome equivalent) was calculated based on the standard curve from the serial dilution of one cDNA from the virus.

## 2′-(4-Methylumbelliferyl)-α-D-N-acetylneuraminic acid

Virus was first diluted at least five-fold in NA buffer (1 per cent BSA + 33 mM MES + 4 mM CaCl_2_, pH = 6.5) to adjust pH and pre-warmed to 37°C. Twenty microliters of the diluted virus was added into a pre-warmed black 96-well plate with 20 μl of pre-warmed MUNANA (200 μM) in NA buffer. The *V*_mean_ of fluorescent signal (ex. 365 nm, em. 450 nm) from 10 to 45 min was obtained by the plate reader (Synergy HTX, BioTek) and used as the NA activity measured by MUNANA assay. The optimal range of reading was predetermined. The NA activity was then normalized by the genome equivalent of the input virus.

## Enzyme-linked lectin assay

The ELLA protocol was based on the previous publication (Gao et al. 2016). Briefly, 96-well plate was coated with fetuin. Virus was diluted in the NA buffer (same with MUNANA), added into the plate, and incubated for 16–18 h. Then, the plate was stained by horseradish peroxidase-conjugated peanut agglutinin (PNA-HRPO). The absorbance at 450nm (A450) was measured by the plate reader after HRPO substrate (1-Step Ultra TMB-ELISA, Thermo Fisher Scientific) was. NA activity was interpolated based on A450 and the standard curve from the serial dilution of a virus and normalized by the genome equivalent of the input virus.

### DMS of HA1

The generation, passaging, and analysis procedures are based on our previous publication. Briefly, we first generated the plasmid library containing all the possible amino acid substitutions on HA1 subunit (D18-R344, H1 numbering from the first amino acid residue) by overlapping PCR (Phusion High-Fidelity DNA Polymerase, Thermo Fisher Scientific) using primers containing the degenerative codon NNK. The fragments were then cloned into pDZ vector that express both the positive and negative sense of virus RNA. The HA1dms plasmid library, along with other seven segments, was co-transfected into HEK-293T cells co-cultured with MDCK-SIAT1. The supernatant was then collected 72 h post-transfection and used to infect MDCK-SIAT1 at MOI of 0.05.

The virus supernatant was harvested 36 h post-infection and prepared for barcode subamplicon sequencing. One hundred forty microliters of virus supernatant was treated with 0.25 μg RNaseA for 30 min in 37°C to remove the extracellular RNA and continued with viral RNA extraction (QIAamp Viral RNA Kits, Qiagen) following the manufacturer’s protocol. The viral RNA product was then treated by DNase to remove the carry-over plasmid DNA (RNeasy Kit, Qiagen). cDNA of the virus genome was generated by reverse transcription with random hexamer as the primer (SuperScript III, Thermo Fisher Scientific). The HA1 subunit was divided into three fragments for PCR (PrimeSTAR Max DNA Polymerase, Takara Bio), and seven Ns and partial sequencing adapters were added in the primer as the barcode. The fragments were purified (PureLink Quick Gel Extraction Kit, Invitrogen) and served as template for the second round PCR to add the full TruSeq adapters and purified (PureLink Quick Gel Extraction Kit, Invitrogen) for NovaSeq. The libraries were pooled, quantitated by qPCR and sequenced on one SP lane for 251 cycles from both ends of the fragments on a NovaSeq 6000 with V1.5 sequencing kits.

The sequencing results were processed by dmstools2 using default parameters (Bloom 2015). In the codon read sheets, codons with more than 20 reads in the WT plasmid were masked to reduce errors and then were translated into amino acid sheets. In the amino acid sheets, mutations with frequency less than 2 × 10^−5^ (∼20 reads) and less than 8 times more in the HA1dms plasmid library than the frequency in the WT plasmid were masked due to insufficient frequency for downstream analysis. The enrichment for each mutation is the fold change between the frequency post-passaging and the frequency in the HA1dms library. The normalized relative fitness score was calculated by the equation below:


$$fitnes{s_{aa}} = \frac{{log(enrichmen{t_{aa}}) - log(enrichmen{t_{nonsense}})}}{{log(enrichmen{t_{silent}}) - log(enrichmen{t_{nonsense}})}}$$

The RBS is defined as follows: residues 133–138, 188–194, 221–228, 98, 153, 183, and 195 (Yang et al. 2010). The surface residues are identified based on the solvent/protein contact surface by the function “FindSurfaceResidues” on PyMOL.

To calculate MAC for a given residue, we first gather the absolute values of coefficients from the linear regression of the normalized relative fitness scores against the NA background activities for the 19 substitutions on the residue. We then calculated the average of the absolute values of coefficients as MAC. This can be expressed as the equation below:


$$MA{C_{residue}} = \frac{\sum\left| {coefficien{t_{aa|residue}}} \right|}{n_{aa}}$$

Here, $coefficien{t_{aa|{\ }residue}}$ is the coefficient of an amino acid substitution on this residue from the linear regression, ${n_{aa}}$ is the total number of valid amino acid substitutions on the residue.

### Antibody expression and purification

Plasmids encoding the heavy chain and light chain of the antibody were transfected at an equimolar ratio into Expi293F cells using ExpiFectamine 293 transfection kit (Gibco) according to the manufacturer’s protocol. Six days post-transfection, cell suspension was harvested and the supernatant obtained through centrifugation at 4500 × *g* and 4°C for 45 min. The supernatant was clarified using a polyethersulfone membrane with a 0.22 μm filter (Millipore).

CH1-XL agarose bead slurry (Thermo Scientific) was washed with MilliQ H_2_O. Washed beads were added into the supernatant and incubated overnight at 4°C with gentle rocking. Then, the supernatant containing unbound antibody was collected and discarded. Beads were washed with PBS. Subsequently, beads were incubated with 60 mM sodium acetate, pH 3.7 for 10 min at room temperature to elute antibody into 1 m Tris-HCl, pH 9.0. The antibody was buffer-exchanged into PBS using an Amicon ultracentrifuge filter with a 30 kDa molecular weight cutoff (Millipore) through four rounds of centrifugation at 3000 × *g* and 4°C for 10 min each. The antibody was further clarified using a Costar Spin-X centrifuge tube with a 0.22 μm cellulose acetate filter (Corning) through centrifugation at 10,000 × *g* and 4°C for 10 min. Antibody concentration was measured with a Nanodrop spectrophotometer.

### Antibody selection

To determine the saturating neutralization concentration, 10^7^ TCID50 of CA09 was incubated with various concentrations of the antibody that was achievable for 30 min in 37°C. The virus–antibody mixture was then used to infect the MDCK-SIAT1 cells in six-well plates for 1 h in 37°C. The monolayer of cells was then washed twice with PBS, and the infectious medium supplemented with the given concentration of antibody was added on top. The supernatant was then collected 24 h post-infection and titered by TCID50 assay. The saturating neutralization concentration was determined as the concentration where adding more antibody would not further decrease the yield of the virus or the highest concentration achievable.

In the selection experiment, the virus population was generated by mixing three independently grown virus stocks to ensure sufficient diversity in the starting population. Similar to the method used to determine the saturating neutralization concentration stated above, 10^7^ TCID50 of the recombinant viruses were neutralized by the antibody in the saturating neutralization concentration and used to infect the cells with six replicates. The virus was passaged to reach sufficient titer (>10^4^ TCID50/ml) for NGS if necessary.

To generate the library for NGS, the virus stock was first treated by RNase to remove the extracellular RNA and continued to viral RNA extraction (QIAamp Viral RNA Kits, Qiagen). Ten microliters of viral RNA was added into 20 μl reverse transcription reaction using MBTuni-12 as the primer and high-fidelity reverse transcriptase (SuperScript III, Thermo Fisher Scientific). The virus genome was further amplified by PCR (Phusion High-Fidelity DNA Polymerase, Thermo Fisher Scientific) with 10 μl cDNA in each 50 μl reaction and MBTuni-12/13 (5′-ACGCGTGATCAGTAGAAACAAGG) as the primers for 25 cycles. The PCR product was then purified (PureLink Quick Gel Extraction Kit, Invitrogen), prepared for NGS by shotgun library preparation (KAPA HyperPrep Kits, Roche), and then sequenced on either MiSeq (MiSeq 500-cycle sequencing kit version 2) or NovaSeq for 250 nt-paired sequencing.

The sequencing results were first parsed by fastp (0.19.5) to remove low-quality reads with a cutoff quality of 28 and minimum length of 100 nt (Chen et al. 2018). After the quality filters, the reads were aligned to the reference sequence by bowtie2 (2.3.5.1) (Langmead and Salzberg 2012) and transform to sorted bam file by SAMtools (1.9) (Danecek et al. 2021). The identity and the frequency of each mutation was identified by ivar (1.3.1) (Grubaugh et al. 2019) using default parameters. To filter the major substitutions selected by the antibody pressure, only substitutions found twice in all the samples and reached 10 per cent in frequency at least once were considered for further analysis.

## Supplementary Material

veae046_Supp

## Data Availability

The raw sequencing data generated in this study were submitted to Sequence Read Archive BioProject ID PRJNA1088522.
