# Correction to: Natural variation in neuraminidase activity influences the evolutionary potential of the seasonal H1N1 lineage hemagglutinin

**DOI:** 10.1093/ve/veae077

**Published:** 2024-09-23

This is a correction to: Tongyu Liu, William K Reiser, Timothy J C Tan, Huibin Lv, Joel Rivera-Cardona, Kyle Heimburger, Nicholas C Wu, Christopher B Brooke, Natural variation in neuraminidase activity influences the evolutionary potential of the seasonal H1N1 lineage hemagglutinin, *Virus Evolution*, Volume 10, Issue 1, 2024, veae046, https://doi.org/10.1093/ve/veae046

In the originally published version of this manuscript, there was an error in the legend of Figure 4, whereby panels (c) and (g) were described as having used antibody 2A05.

The legend should instead have stated that only (c) refers to 2A05, while (g) should have indicated 2C05.

This has been corrected.

