# Supplementary material for: Natural variation in neuraminidase activity influences the evolutionary potential of the seasonal H1N1 lineage hemagglutinin
Source: Virus Evol. 2024 Jun 19;10(1):veae046. doi: 10.1093/ve/veae046 (PMC11196192; doi:10.1093/ve/veae046)
Supplement: veae046_Supp [file veae046_supp.zip › suppl_data/supplement figures.pdf]

Supplementary Figures

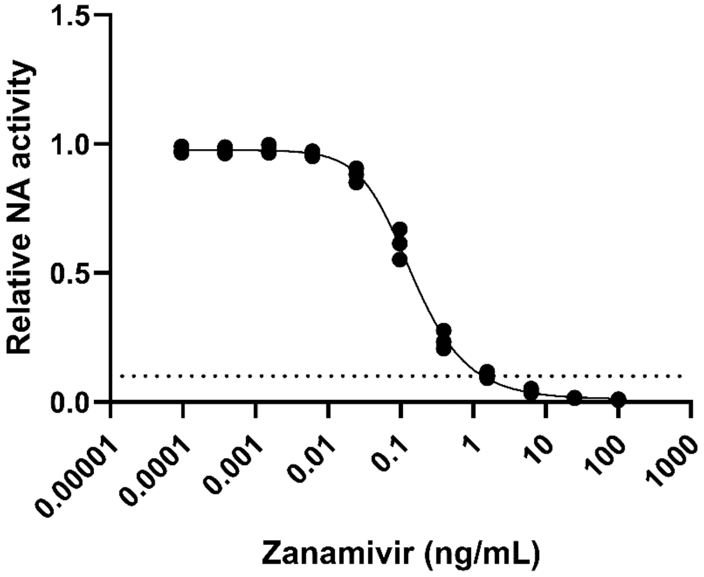

Figure S1: Inhibition curve of zanamivir against CA09 virus measured by MUNANA assay. *Nonlinear curve fits by asymmetric sigmoidal in GraphPad Prism, dot line indicates 90% inhibition of NA activity, n = 3.*

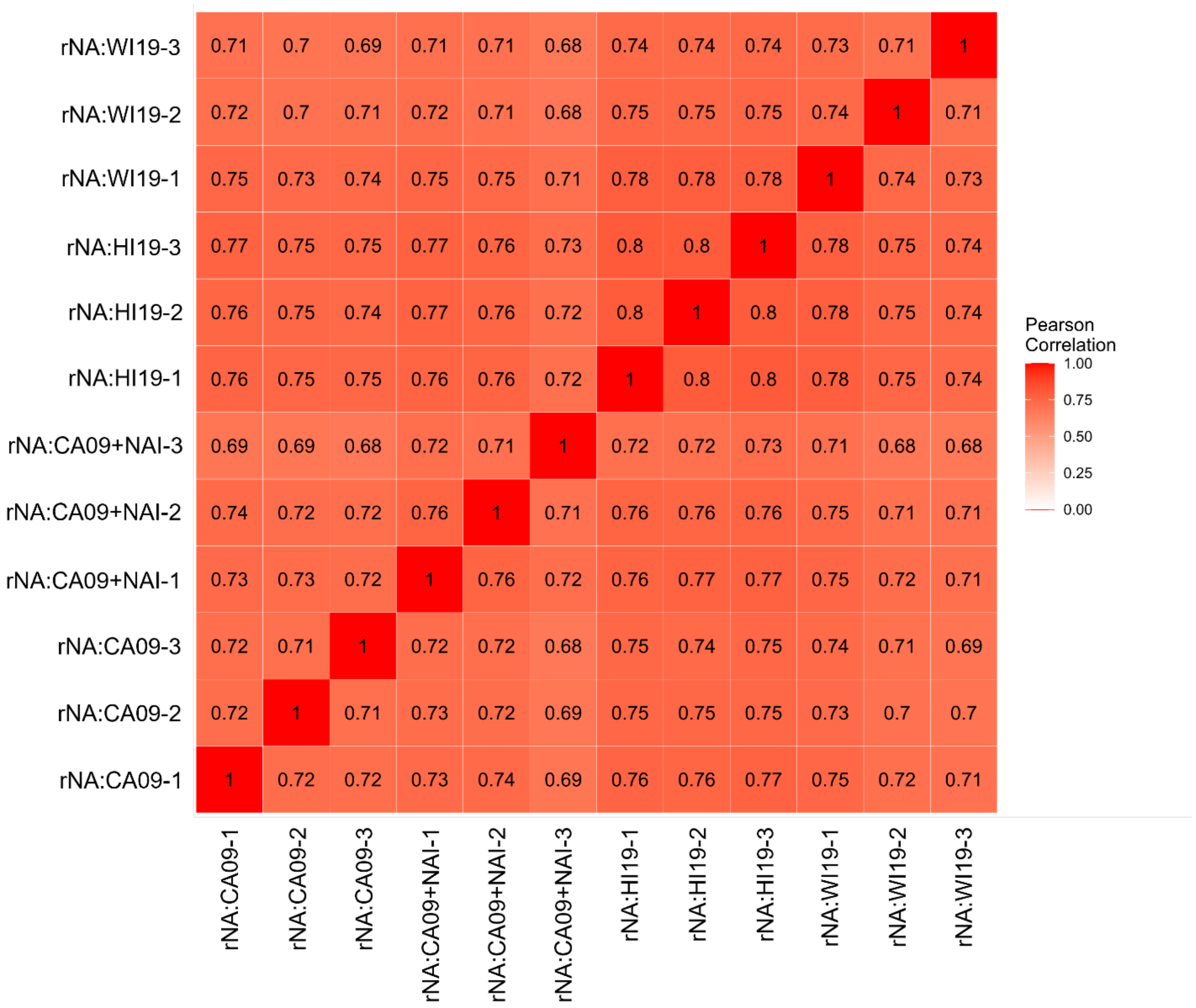

Figure S2: Pearson correlations of normalized relative fitness score between samples in deep mutational scanning.

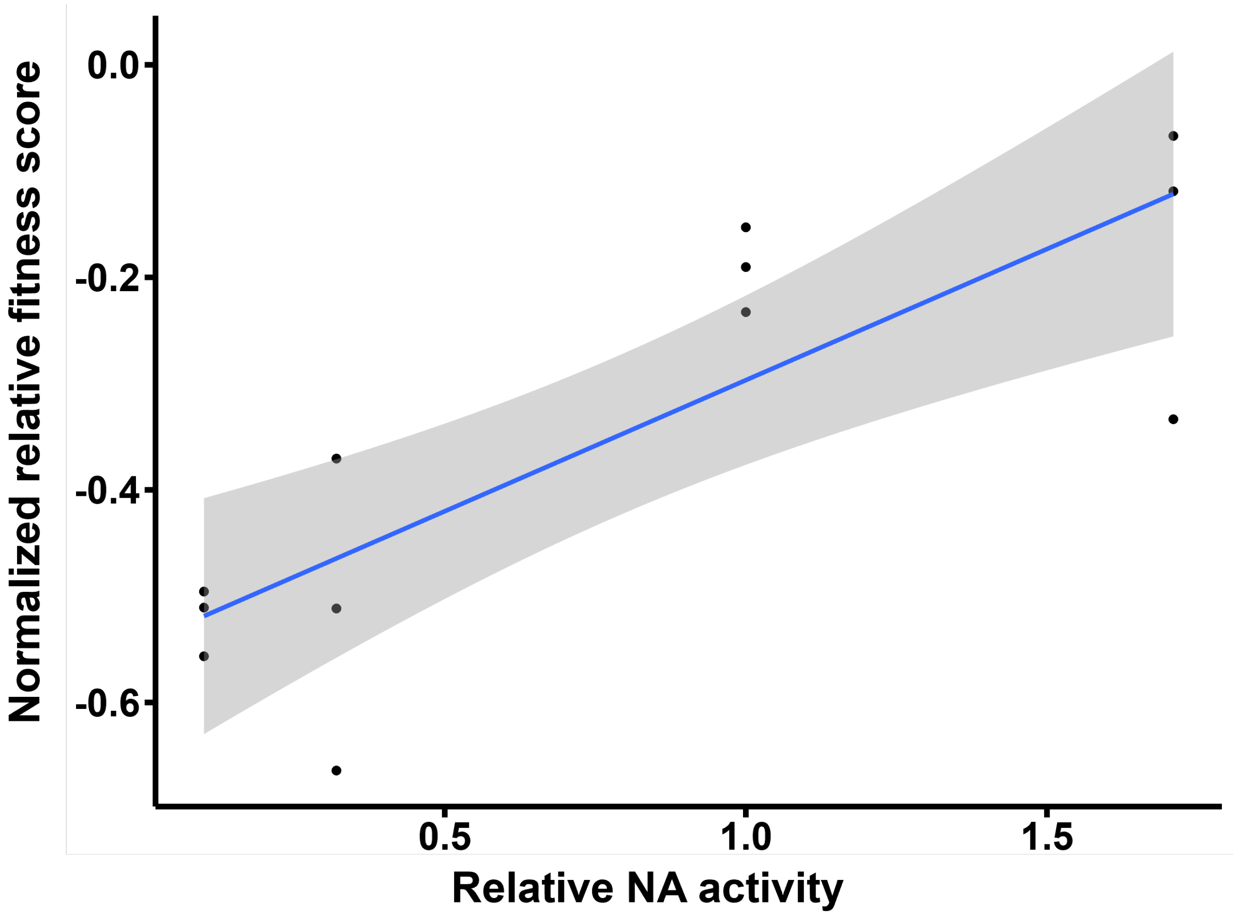

Figure S3: Example of linear regression of normalized relative fitness score against the relative NA activity for a given substitution. *Dots represent the normalized fitness scores measured in the given NA activity backgrounds in triplicates, blue line indicates the best-fit line by linear regression (lm() in R), gray area is the 95% interval of the regression.*

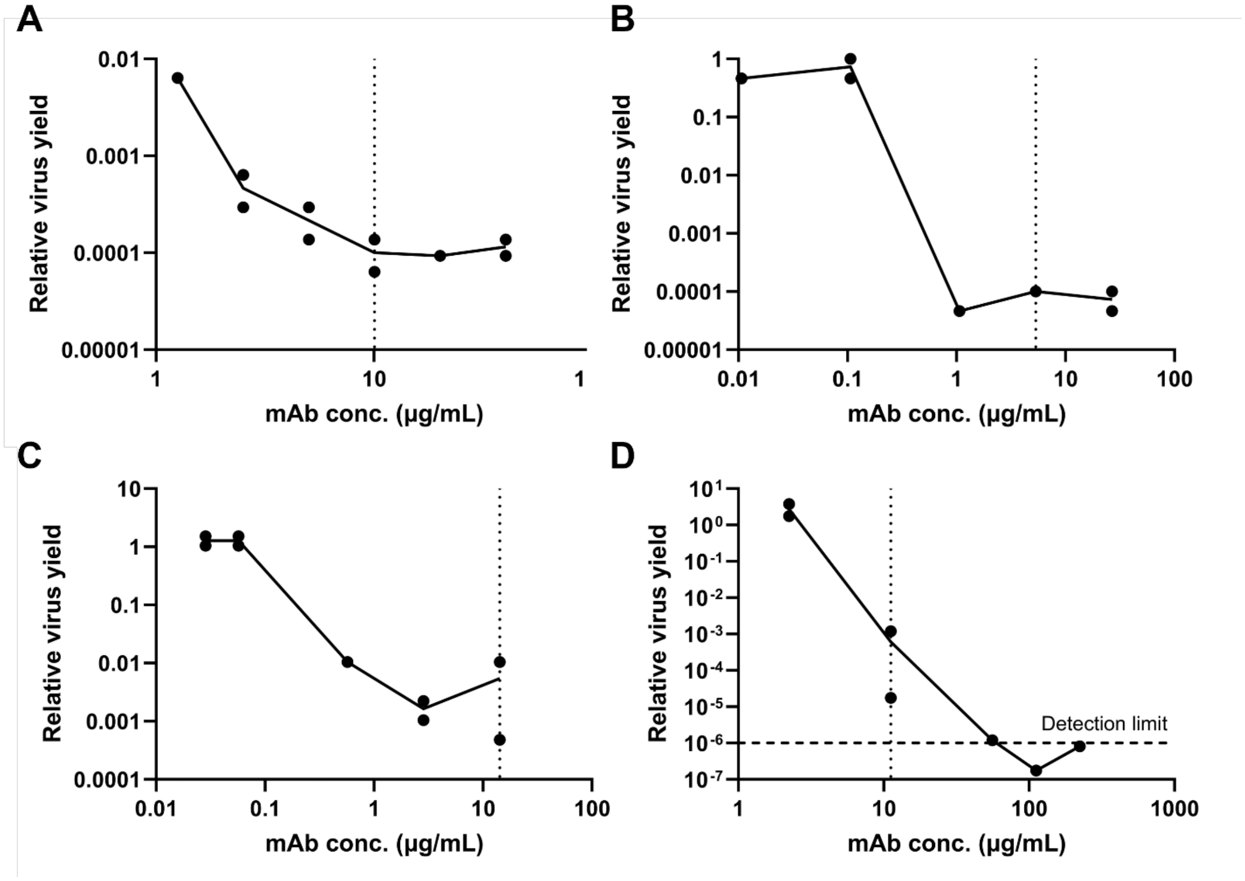

Figure S4: Saturated neutralization concentrations for the monoclonal antibodies used in this study. *10^7 TCID50 of rNA:CA09 was neutralized with (A) EM4-C04, (B) 2A05, (C) 2C05 and (D) CR9114 in the given concentration and infected the cells. Virus supernatants were collected 24 hours post infection (n = 2) and measured by TCID50 assay. Relative virus yield was normalized to the no antibody control group. Dot lines indicate the concentration used in selection.*

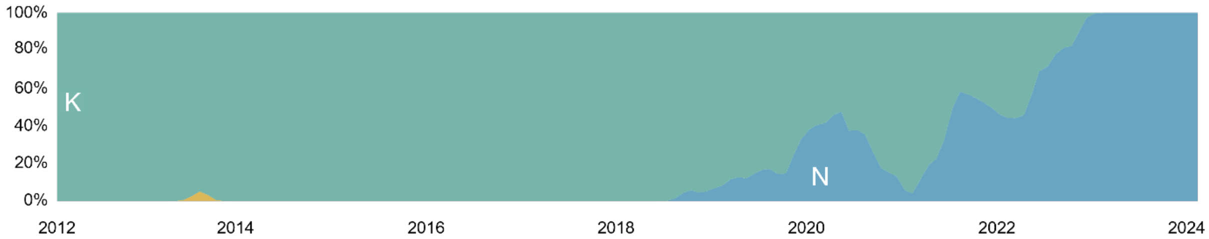

Figure S5: Normalized frequencies composition of pdmH1N1 HA position 133a over the years *colored by the amino acid: lysine(K, green), asparagine (N, blue), arginine (R, yellow) from 1472 genomes samples between February 2012 to February 2024 (Screenshot taken from Nextstrain[34,60]).*

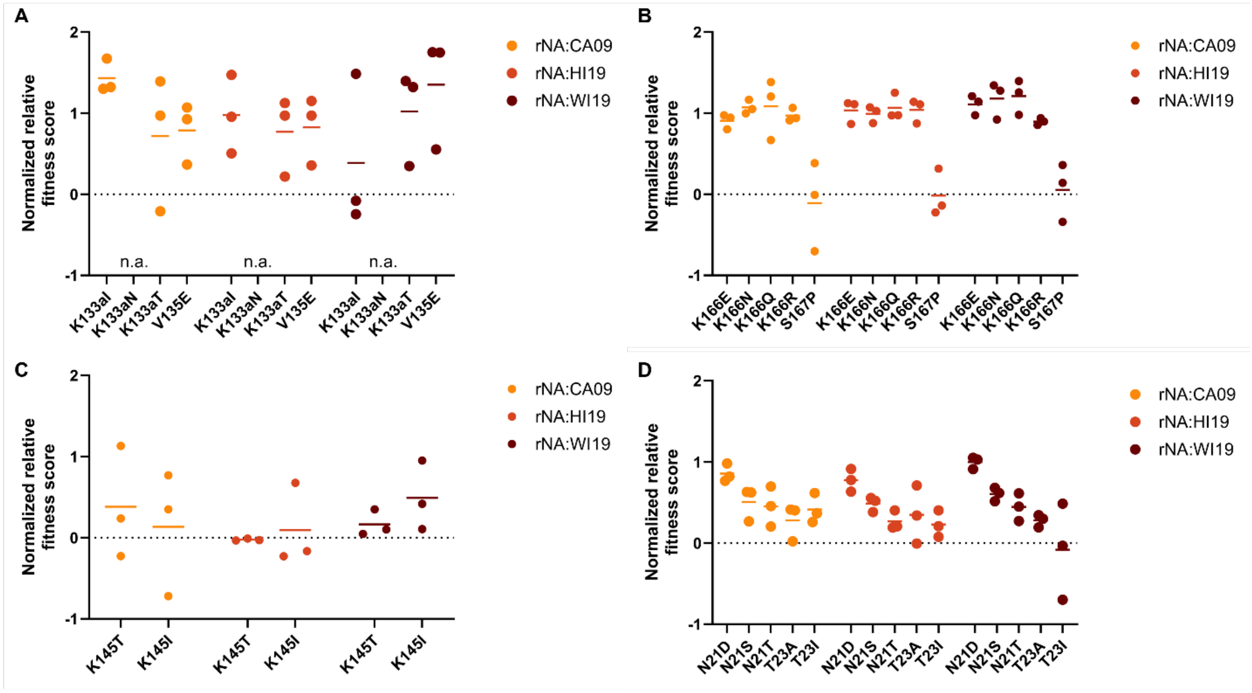

Figure S6: The normalized relative fitness score in deep mutational scanning of escape variants found in antibody selection *with (A) EM4-C04, (B) 2A05, (C) 2C05 and (D) CR9114 (excluding HA2 residues).*
